# High-sensitivity whole-mount in situ Hybridization of Mouse Oocytes and Embryos Visualizes the Super-resolution Structures and Distributions of mRNA Molecules

**DOI:** 10.1186/s12575-024-00250-5

**Published:** 2024-07-10

**Authors:** Takahiro Sanada, Tomoya Kotani

**Affiliations:** 1 Biosystems Science Course, Graduate School of Life Science, Hokkaido University, Sapporo, 060-0810 Japan; 2 Department of Biological Sciences, Faculty of Science, Hokkaido University, North 10 West 8, Sapporo, 060-0810 Hokkaido Japan

**Keywords:** Mammal, Oocyte, Embryo, Maternal mRNA, in situ hybridization, Super-resolution microscopy

## Abstract

Mammalian oocytes accumulate more than ten thousand mRNAs, of which three to four thousand mRNAs are translationally repressed. The timings and sites of translational activation of these dormant mRNAs are crucial for promoting oocyte maturation and embryonic development. How these mRNAs are accumulated and distributed in oocytes is therefore a fundamental issue to be explored. A method that enables visualization of mRNA molecules with high resolution in a simple manner would be valuable for understanding how oocytes accumulate and regulate the dormant mRNAs. We have developed a highly sensitive whole-mount in situ hybridization method using in vitro-synthesized RNA probes and the tyramide signal amplification (TSA) system optimized for mouse oocytes and embryos. By using this method, *Pou5f1/Oct4*, *Emi2*, and cyclin B1 mRNAs were detected in immature oocytes and 2-cell stage embryos. Confocal microscopy showed that these mRNAs formed granular structures in the oocyte cytoplasm. The structures of *Pou5f1/Oct4* and cyclin B1 mRNAs persisted in 2-cell stage embryos. *Pou5f1/Oct4* RNA granules exhibited a solid-like property in immature oocytes and became liquid-like droplets in 2-cell stage embryos. Double-staining of cyclin B1 mRNA with *Emi2* or *Pou5f1/Oct4* mRNA revealed that these mRNAs were distributed as different RNA granules without overlapping each other and that the size of cyclin B1 RNA granules tended to be larger than that of *Emi2* RNA granules. The structures and distribution patterns of these mRNAs were further analyzed by N-SIM super-resolution microscopy. This analysis revealed that the large-sized RNA granules consist of many small-sized granules, suggesting the accumulation and regulation of dormant mRNAs as basal-sized RNA granules. The method established in this study can easily visualize the structure and distribution of mRNAs accumulated in mammalian oocytes and embryos with high sensitivity and super-resolution. This method is useful for investigating the cellular and molecular mechanisms of translational control of mRNAs by which maturation and early developmental processes are promoted.

**Supplementary Information:**

The online version contains supplementary material available at 10.1186/s12575-024-00250-5.

## Introduction

The initiation and early processes of mammalian development are driven by maternally inherited materials in oocytes. Since transcription is quiescent from the initiation of oocyte maturation until the 2-cell stage in mice [1] and the 4- to 8-cell stage in humans [2], proteins promoting oocyte maturation and early development are synthesized from mRNAs accumulated in oocytes. Due to recent technical advances, it has been revealed that mammalian oocytes accumulate more than 10,000 mRNAs during oogenesis. Of these mRNAs, approximately 1,000 to 2,000 mRNAs are translated during oocyte maturation and 2,000 to 3,000 mRNAs are translated after fertilization [3–7]. Although these comprehensive studies have unveiled the timings of translation of bulk mRNAs, how the individual mRNAs are accumulated in oocytes and how they are regulated during oocyte maturation and early development remain unclear.

The translational activation of several mRNAs that is required for oocyte maturation has been investigated over the past four decades. Fully grown immature oocytes arrest meiotic cell cycles at prophase of meiosis I and resume meiosis in response to specific signals such as hormones. The synthesis of Cyclin B1 protein from stored cyclin B1 mRNA promotes the activation of maturation/M-phase-promoting factor (MPF), leading to the cytoplasmic and nucleic maturation of oocytes [8, 9]. Endogenous meiotic inhibitor 2 (Emi2) protein, also known as F-box protein 43 (Fbxo43), has been identified as an inhibitor of anaphase-promoting complexes/cyclosome (APC/C) in mouse and *Xenopus* oocytes [10–12]. After resumption of meiosis, Emi2 is synthesized from stored *Emi2* mRNA at the end of meiosis I, preventing the complete degradation of Cyclin B1 in the transition from meiosis I to meiosis II [13] and resulting in the arrest of meiosis at metaphase II [11].

A recent study demonstrated that Smarcd2 and Cyclin T2 are synthesized from the mRNAs stored in eggs after fertilization and that the synthesized proteins are critical for zygotic genome activation [6]. Pou5f1/Oct4 protein has been identified as a germline-specific transcription factor that is essential for mammalian development [14–16]. A reduction and an increase of Pou5f1/Oct4 synthesis impaired the progression of normal development in mouse and human embryos [17–19]. All of those studies demonstrated the importance of temporal translation of individual mRNAs that are stored in oocytes as dormant forms for the proper promotion of oocyte maturation and early development. Extensive biochemical studies have shown that the temporal regulation of mRNA translation is driven by *cis*-acting elements in dormant mRNAs and *trans*-acting factors expressed in oocytes [20]. However, how the dormant mRNAs are accumulated and regulated in the cytoplasm of oocytes and embryonic cells remains to be investigated. By developing a method for highly sensitive paraffin section in situ hybridization, we have revealed that dormant cyclin B1, *Emi2*, and *Pou5f1/Oct4* mRNAs are accumulated in the oocyte cytoplasm as granular structures in mice and zebrafish [13, 21–26]. Although those studies provided basal findings for the accumulation and regulation of dormant mRNAs, the sensitivity for detecting mRNAs in mouse oocytes and embryos was not sufficiently high and the method required multiple steps for detecting mRNA molecules. In addition, all of the steps require large amounts of reagents. Moreover, mammalian oocytes and embryos isolated from ovaries and oviducts cannot be directly embedded in paraffin due to their small sizes.

Recent studies have shown that a single molecule RNA fluorescent in situ hybridization (smRNA-FISH) with the RNA-scope technique enables detection of mRNAs in mammalian oocytes and embryos with high sensitivity in whole-mount samples [27–29]. In these methods, mRNAs are detected by the hybridization of Z-shaped DNA probes consisting of 50–60 bases, of which 20–25 bases on one side are complementary to sequences of the specific transcripts and 14 bases on the other side are complementary to pre-amplifier DNA sequences. One pair of the Z probes enables amplification of signals through hybridization of pre-amplifier DNA followed by hybridization of amplifier DNAs and binding of fluorophores. Generally, 10–20 pairs of the Z probes are used for detecting a specific transcript. Although the Z probes are commercially available, preparation of them for detecting a large number of different target mRNAs is resource intensive. In addition, three round hybridization steps are required for amplification of signals. These methods are costly and require much effort for detecting mRNAs. The first in situ hybridization method was developed in the 1970s with in vitro-synthesized RNA probes [30]. Subsequent modifications of this method such as the use of digoxigenin (DIG) and Fluorescein-conjugated RNA probes and the amplification of RNA-probe signals using the TSA system enabled detection of mRNAs with high sensitivity and high resolution in a convenient and cost-effective manner [31–35].

In this study, we established a whole-mount in situ hybridization method with RNA probes and the TSA system for detecting mRNAs in mammalian oocytes and embryos in a simple and cost-effective manner with small amounts of reagents. Using this method, we analyzed the distribution patterns of *Pou5f1/Oct4*, *Emi2* and cyclin B1 mRNAs in oocytes and embryos with high sensitivity and super-resolution. This method will contribute to an understanding of the regulation of mRNAs that have accumulated in mammalian oocytes and embryos.

## Materials and Methods

### Isolation of Oocytes and Embryos

ICR mice were maintained on a 14-h light/10-h dark cycle at 25˚C with free access to food and water. Fully grown oocytes (GV-stage) of more than 50 μm in diameter were isolated by puncturing ovaries from 8-week-old females with a needle in M2 medium (94.7 mM NaCl, 4.8 mM KCl, 1.7 mM CaCl_2_, 1.2 mM KH_2_PO_4_, 1.2 mM MgSO_4_, 4.2 mM NaHCO_3_, 20.9 mM HEPES, 23.3 mM sodium lactate, 0.3 mM sodium pyruvate, 5.6 mM glucose, 0.1 mM gentamicin, 0.01 mg/ml phenol red, 4 mg/ml BSA; pH7.2-7.4) containing 10 µM milrinone as an inhibitor for the resumption of meiosis (M2+). These females were not injected with PMSG. Embryos were obtained by in vitro fertilization as follows. To collect ovulated mature oocytes, females were injected with 5 IU hCG 48 h after injection with 5 IU PMSG. Sperms were isolated from the epididymis tail from 8-week-old males with a needle in a petri dish filled with paraffin oil (Nacalai Tesque) and put into human tubal fluid (HTF) medium (102 mM NaCl, 4.7 mM KCl, 2 mM CaCl_2_, 0.37 mM KH_2_PO_4_, 0.2 mM MgSO_4_, 25 mM NaHCO_3_, 21.4 mM sodium lactate, 0.33 mM sodium pyruvate, 2.8 mM glucose, 0.1 mM gentamicin, 0.01 mg/ml phenol red, 4 mg/ml BSA) placed in the same dish and filled with paraffin oil. After the incubation of sperms with HTF medium at 37℃ for 2 h in 5% CO_2_/95% air, mature oocytes were isolated from oviducts of females in HTF medium. Then the sperms were added to the medium, and the oocytes were further incubated for 6 h. The fertilized eggs were washed with M16 + EDTA medium (94.7 mM NaCl, 4.8 mM KCl, 1.7 mM CaCl_2_, 1.2 mM KH_2_PO_4_, 1.2 mM MgSO_4_, 25 mM NaHCO_3_, 23.3 mM sodium lactate, 0.3 mM sodium pyruvate, 5.6 mM glucose, 0.1 mM gentamicin, 0.01 mg/ml phenol red, 4 mg/ml BSA) containing 1 mM EDTA. The zygotes were incubated with M16 + EDTA at 37˚C for 18 h in 5% CO_2_/95% air.

### Solution Preparation

For in situ hybridization, stock solutions were prepared as follows. 20x saline-sodium citrate (20x SSC: 3 M NaCl, 200 mM sodium citrate; pH 7.0) was autoclaved and stored. Torula RNA (Sigma) was dissolved in DDW at 50˚C and purified by phenol-, phenol-chloroform-, and chloroform-extraction. After ethanol precipitation, the RNAs were dissolved in DDW (10 mg/ml) and stored at -20˚C. 20x Denhardt’ solution was prepared by dissolving Ficol-400 (0.4%), polyvinylpyrrolidone (0.4%) and bovine serum albumin (0.4%) in DDW and was stored at -20˚C.

### Probe Preparation

DIG- and fluorescein-labeled RNA probes were prepared by in vitro transcription with RNA polymerases and plasmid vectors containing target transcript sequences. One µg of linearized plasmid DNA was used as a template, and RNA probes were synthesized with SP6 or T7 RNA polymerase (Roche) and DIG or Fluorescein RNA Labeling Mix (Roche) for 2–3 h at 37˚C. After precipitation with isopropanol and sodium acetate, the RNA probes were dissolved in DDW. After determining the concentrations, the RNA probes were diluted (50 ng/µl) with probe dilution buffer (50% formamide, 5x SSC, 0.1% Tween-20) containing torula RNA (5 mg/ml) and were stored at -20˚C.

In this study, we prepared 6 DIG-labeled antisense and sense RNA probes for the full lengths of *Pou5f1/Oct4*, *Emi2*, and cyclin B1 transcripts. These three mRNAs were shown to be translationally repressed after transcription during oogenesis and translated at different timings. The translation of *Pou5f1/Oct4* mRNA is activated after fertilization [25] and that of *Emi2* and cyclin B1 mRNAs is activated at distinct timings after initiation of oocyte maturation [13, 24]. In addition, 2 Fluorescein-labeled antisense and sense RNA probes for the full length of cyclin B1 were prepared. The sequences of all of the transcripts used for making RNA probes were shown in our previous studies [13, 23]. To detect the specific transcript, we used the full lengths of transcripts. In general, more than 500 base sequences are used for detection of the specific transcript.

### Fixation and Permeabilization of Oocytes and Embryos

**Fig. 1:**
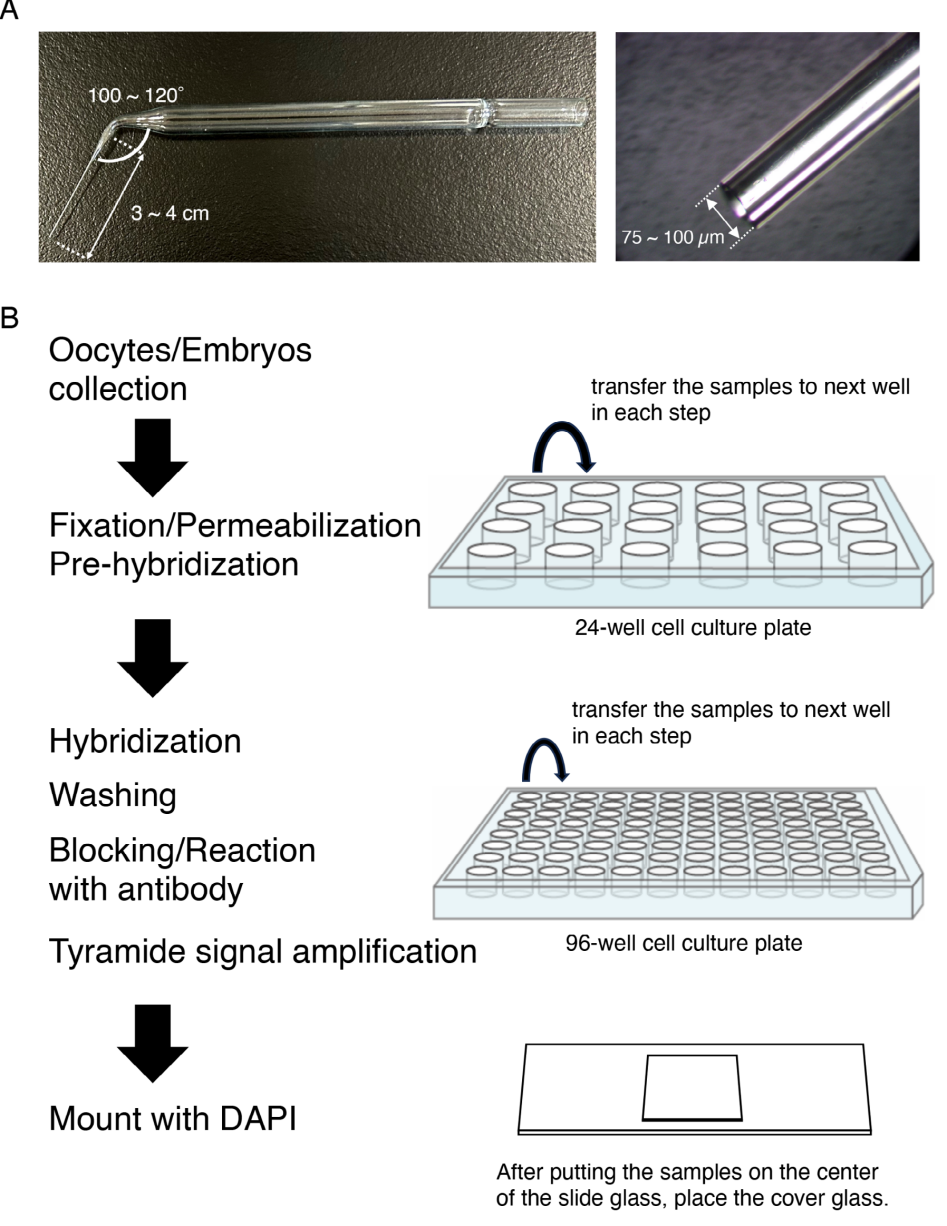
Schematic view of the procedure for whole-mount in situ hybridization optimized for mouse oocytes and embryos. **A** The structure of glass pipettes used in the procedure. **B** After the oocytes/embryos had been collected in a 24-well cell culture plate, the samples were fixed with 4% PFA/PBS, permeabilized with 2% Triton X-100, and incubated with 66% formamide/10% SSC (pre-hybridization). A 24-well cell culture plate was used in these steps. Then the oocytes/embryos were transferred to a 96-well cell culture plate and hybridized with probes of target RNAs at 45˚C overnight. The samples were washed with a series of SSC and incubated with blocking buffer. Then anti-DIG or Fluorescein antibody conjugated with horseradish peroxidase (HRP) was added, and signals were amplified with the TSA system. A 96-well cell culture plate was used in these steps. Finally, the samples were mounted on a slide glass with mounting medium with DAPI

After being isolated, the oocytes and embryos were washed with isolation buffer [0.004% polyvinyl pyrrolidone K30 (Wako Pure Chemical Industries, Ltd.), 0.1% RNase inhibitor (Takara) in PBS] and fixed with 4% paraformaldehyde in PBS (4% PFA/PBS) for 30 min at room temperature in a 24-well cell culture plate (Fig. 1B). The addition of polyvinyl pyrrolidone K30 enables immediate sinkage of oocytes and embryos in PFA/PBS. After fixation, the oocytes and embryos were permeabilized with permeabilization solution [2% Triton-X100, 1 mM DTT, 0.1% RNase inhibitor (Takara) in PBS] for 20 min at room temperature. This treatment enables omission of proteinase treatment. The addition of 1 mM DTT stabilizes the RNase inhibitor.

### Hybridization

After permeabilization, the samples were incubated with 66% formamide in 2x SSC for 1 h at room temperature. The oocytes and embryos were hybridized with a mixture of 1 ng/µl of Fluorescein and/or DIG-labeled RNA probes in hybridization mix solution (70% formamide, 20 mM Tris-HCl; pH 8.0, 2.5 mM EDTA, 1x Denhardt’s solution, 30 mM NaCl, 1 mg/ml torula RNA) containing 10% dextran sulfate at 45˚C for 12–16 h. The concentration of the RNA probes (1 ng/µl) resulted in highly specific and less background signals. From this step, a 96-well cell culture plate with a lid was used (Fig. 1B). The samples were then washed with 50% formamide in 2x SSC at 50˚C for 30 min. After incubation with Tris-NaCl-EDTA (TNE) buffer (10 mM Tris, 500 mM NaCl, 1 mM EDTA; pH 7.5) at 37˚C for 10 min, the samples were incubated with 20 µg/ml RNase A (Sigma) in TNE at 37˚C for 30 min to reduce nonspecific background signals [36]. Then the samples were washed with TNE at 37˚C for 10 min, with 2x SSC at 50˚C for 20 min, and with 0.2x SSC at 50˚C for 20 min. Incubation with 0.2x SSC was performed twice. To prevent the oocytes and embryos from sticking to the walls of the culture plate wells, 0.1% Tween-20 was added to the SSC series. Next, the samples were incubated with Tris-NaCl-Tween (TNT) buffer (100 mM Tris, 150 mM NaCl, 0.5% Tween-20; pH 7.5) at room temperature for 5 min. The samples were then treated with 1% H_2_O_2_ in 1x PBS containing 0.1% Tween-20 at room temperature for 60 min to inactivate endogenous peroxidase.

### Detection of DIG- and Fluorescein-labeled RNA Probes

Single in situ hybridization of mRNA encoding *Pou5f1/Oct4*, *Emi2*, and cyclin B1 in mouse oocytes and embryos was performed as follows. After hybridization with the DIG-labeled probes described above, the samples were blocked with blocking buffer [0.5% blocking reagent (PerkinElmer, Inc), 100 mM Tris, 150 mM NaCl; pH 7.5] at room temperature for 30 min. Then the samples were treated with anti-DIG-horseradish peroxidase (HRP) antibody (Roche) (1:500 dilution in blocking buffer). After being washed with TNT three times, the samples were treated with tyramide-Fluorescein (Akoya Bio, Inc) [1:50 dilution in 1x Plus Amplification Diluent (Akoya Bio. Inc), followed by dilution with an equal volume of DDW] at room temperature for 15–30 min. The reaction for 15 min resulted in highly specific and less background signals. The incubation time could be extended when the signals were weak. Finally, the samples were washed with TNT three times.

Double in situ hybridization of mRNA encoding cyclin B1 and *Pou5f1/Oct4* or *Emi2* in mouse oocytes and embryos was performed as follows. After hybridization with the Fluorescein- and DIG-labeled probes described above, the samples were incubated with blocking buffer at room temperature for 30 min. Then the samples were treated with anti-Fluorescein-HRP antibody (Roche) (1:500 dilution in blocking buffer). After being washed with TNT three times, the samples were treated with tyramide-Cy3 (Akoya Bio. Inc) (1:50 dilution in 1x Plus Amplification Diluent, followed by dilution with an equal volume of DDW) at room temperature for 15 min. After being washed with TNT three times, the samples were treated with 1% H_2_O_2_ in PBS containing 0.1% Tween-20 at room temperature for 60 min to inactivate horseradish peroxidase. The samples were incubated with blocking buffer at room temperature for 30 min. The samples were then treated with anti-DIG-HRP antibody (1:500 dilution in blocking buffer). After being washed with TNT three times, the samples were treated with tyramide-Fluorescein (1:50 dilution in 1x Plus Amplification Diluent, followed by dilution with an equal volume of DDW) at room temperature for 15–30 min. Finally, the samples were washed with TNT three times.

### DAPI Staining and Mounting

To detect nuclei or chromosomes, the samples were mounted with VECTASHIELD Mounting Medium with DAPI (Funakoshi) on a slide glass (Fig. 1B). After being mounted, the samples were observed under an LSM980 confocal microscope (Carl Zeiss) using a Plan Apochromat 63x/1.4 NA oil differential interference contrast lens (Carl Zeiss) and under an N-SIM super-resolution microscope (Nikon) using a CFI Apochromat TIRF 100xC oil lens (Nikon).

### Injection of siRNAs

To knock down *Pou5f1/Oct4*, *Emi2* or cyclin B1 mRNA by siRNA, 10 pl of 25 µM *Pou5f1/Oct4*, *Emi2* or cyclin B1 siRNA (GeneDesign, Inc.; sequences shown in Fig. S2A) was injected into GV-stage oocytes using an IM-9B microinjector (Narishige) under a Dmi8 microscope (Leica). After incubation at 37˚C in M2 + overnight, the oocytes were fixed with 4% PFA for 30 min at room temperature and used for whole-mount in situ hybridization.

### Hexanediol Treatment

To dissolve liquid droplets, isolated mouse oocytes and embryos were treated with 1,6-hexanediol (50 mg/ml in M2 + medium) for 20 min at 37˚C. As a control, oocytes and embryos were treated with 1,2,6-hexanetriol (50 mg/ml in M2 + medium) for 20 min at 37˚C. After fixation and permeabilization, the oocytes and embryos were analyzed by whole-mount in situ hybridization.

### Section in situ Hybridization

Section in situ hybridization of mouse oocytes was performed according to a previous study [23]. In brief, the fixed ovaries were dehydrated, embedded in paraffin, and cut into 10-µm-thick sections. The sections of mouse ovaries were hybridized with a hybridization mix containing 1 ng/µl of DIG- and Fluorescein-labeled RNA probes at 45˚C overnight. After hybridization and washing, the samples were incubated with anti-Fluorescein-HRP antibody (1:500 dilution) at room temperature for 30 min. After reaction with tyramide-Cy3 at room temperature for 20 min, the samples were incubated with 1% H_2_O_2_ in PBS at room temperature for 30 min in order to inactivate HRP. Then the samples were incubated with anti-DIG-HRP antibody (1:500 dilution) at room temperature for 30 min. The reaction with tyramide-Fluorescein was performed at room temperature for 30 min. To detect nuclei, the sections were incubated with 10 µg/ml Hoechst 33258 (Sigma) at room temperature for 20 min and observed under the LSM 980 confocal microscope.

## Results

### Detection of *Pou5f1*/*Oct4*, *Emi2* and Cyclin B1 mRNAs by whole-mount Fluorescence in situ Hybridization of Mouse Oocytes and Embryos

We previously developed a high-sensitive and high-resolution section in situ hybridization method using in vitro-synthesized RNA probes and the TSA system [21, 23, 25]. In this method, organs and tissues such as zebrafish and mouse ovaries are fixed, dehydrated, and embedded in paraffin, followed by making thin sections, rehydration, and probe hybridization. This method enabled detection of the distribution of mRNA molecules in single cells of organs. However, it requires laborious efforts and large amounts of reagents to operate samples on the glass slides. In addition, small-size samples such as mammalian oocytes and embryos cannot be embedded in paraffin due to the inability to handle them in paraffin. Although an in situ hybridization method for mammalian oocytes and embryos without making paraffin sections would be useful, isolated mammalian oocytes and embryos are very fragile throughout all steps, particularly in the steps for hybridization and washing out non-hybridized RNA probes. To overcome these problems, we remarkably modified the procedure of our previous method and developed a whole-mount in situ hybridization method that is optimized for mammalian oocytes and embryos.

We first changed the fixation condition: the percentage of paraformaldehyde was increased up to 4%, which enables stable handling of mouse oocytes and embryos in all steps. Second, we modified the structure of glass pipettes by bending the bottom of the tip like an L-shape (Fig. 1A), which allows mild pipetting of the oocytes and embryos. Third, we added a detergent (0.1% Tween-20) to SSC wash buffers, which enables maintenance of the round shapes of oocytes and embryos without sticking to the walls of culture plate wells. All of these modifications enabled easy and safe handling of mouse oocytes and embryos. Since whole-mount samples tend to maintain endogenous peroxidase activities, which significantly increase the background of TSA signals, we treated the samples with 1% H_2_O_2_ in PBS containing 0.1% Tween-20 for 30 min to inactivate the endogenous peroxidase. Throughout all steps, we carefully placed the oocytes and embryos on the bottom of the plate well using the L-shaped glass pipettes (Fig. 1B).

**Fig. 2:**
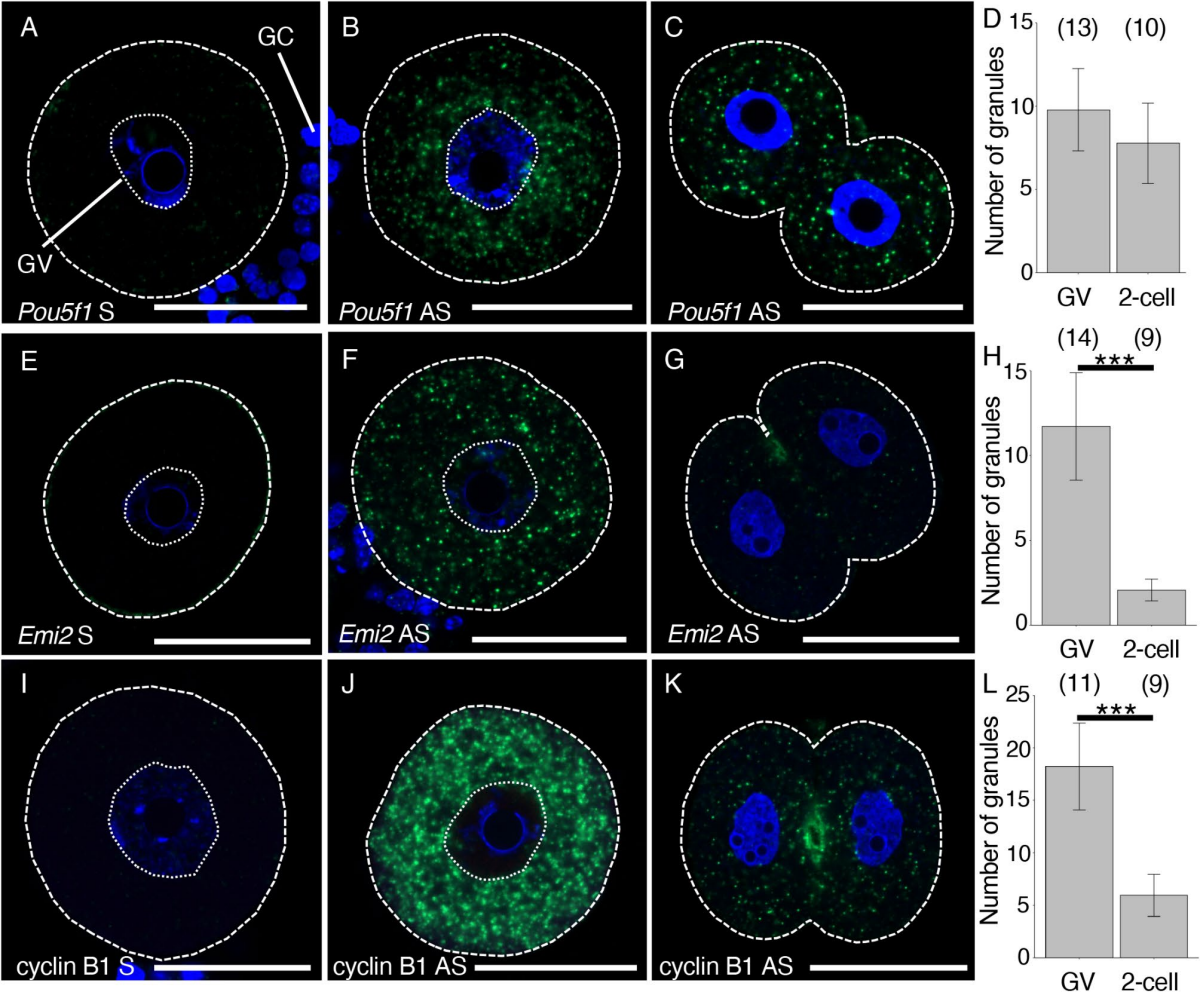
in situ hybridization of *Pou5f1/Oct4*, *Emi2* and cyclin B1 mRNAs in mouse oocytes and embryos. **A-C** Detection of *Pou5f1/Oct4* mRNA in immature oocytes (A-B) and 2-cell stage embryos (C) hybridized with the sense (A) or antisense (B-C) RNA probe. DNA is shown in blue. **D** The numbers of *Pou5f3/Oct4* RNA granules per 100 µm^2^ in individual oocytes and 2-cell stage embryos were counted (means ± standard deviations). Results from three independent experiments were summarized. **E-G** Detection of *Emi2* mRNA in immature oocytes (E-F) and 2-cell stage embryos (G) hybridized with the sense (E) or antisense (F-G) RNA probe. DNA is shown in blue. **H** The numbers of *Emi2* RNA granules per 100 µm^2^ in individual oocytes and 2-cell stage embryos were counted. Results from three independent experiments were summarized. **I-K** Detection of cyclin B1 mRNA in immature oocytes (I-J) and 2-cell stage embryos (K) hybridized with the sense (I) or antisense (J-K) RNA probe. DNA is shown in blue. **L** The numbers of cyclin B1 RNA granules per 100 µm^2^ in individual oocytes and 2-cell stage embryos were counted. Results from three independent experiments were summarized. The numbers in parentheses indicate the total numbers of oocytes and embryos analyzed. Statistical significance was analyzed by Student’s *t*-test. ***p < 0.001. Similar results were obtained from five independent experiments. GC, granulosa cell; GV, germinal vesicle. Bars, 50 μm

After hybridization with DIG-labeled RNA probes and amplification of signals with the TSA system, we observed the mouse oocytes and embryos under a confocal microscope. No signal was detected in oocytes hybridized with the sense *Pou5f1/Oct4* RNA probe (Fig. 2A). In contrast, bright signals were detected in the cytoplasm of mouse oocytes and 2-cell stage embryos hybridized with the antisense *Pou5f1/Oct4* RNA probe (Fig. 2B-D). All images in this study represent the average of samples analyzed. The signals of *Pou5f1/Oct4* mRNA appeared to represent granular structures in the cytoplasm of oocytes and 2-cell stage embryos. In immature oocytes, many of the signals of *Pou5f1/Oct4* mRNA appeared to congregate around the germinal vesicle (GV) (Fig. 2B and Fig. S1A). In contrast, the signals appeared to be distributed all over the cytoplasm in 2-cell stage embryos (Fig. 2C). The antisense *Pou5f1/Oct4* RNA probe indeed detected the *Pou5f1/Oct4* mRNA molecules because almost all signals disappeared when the *Pou5f1/Oct4* mRNA was knocked down by siRNA (Fig. S2A-E).

The accumulation and distribution of *Emi2* and cyclin B1 mRNAs were similarly examined. No signal was detected in oocytes hybridized with the sense *Emi2* RNA probe, whereas bright signals were observed in the cytoplasm of oocytes hybridized with the antisense *Emi2* RNA probe (Fig. 2E and F). The signals with the antisense probe were significantly reduced when the *Emi2* mRNA was knocked down by siRNA (Fig. S2A and F-I), confirming the specificity of signals. The *Emi2* mRNA was distributed throughout the oocyte cytoplasm (Fig. 2F and Fig. S1A). These signals disappeared in 2-cell stage embryos (Fig. 2G and H), consistent with the disappearance of *Emi2/Fbxo43* mRNA after fertilization in RNA-seq analysis [6, 37]. No signal was detected in oocytes hybridized with the sense cyclin B1 RNA probe, whereas very bright signals were observed in oocytes hybridized with the antisense cyclin B1 RNA probe (Fig. 2I and J and Fig. S1B). These signals were significantly reduced when the cyclin B1 mRNA was knocked down by siRNA (Fig. S2A and J-M), confirming the specificity of signals. The signals of cyclin B1 mRNA resembled granule structures and were also detected in the cytoplasm of 2-cell stage embryos, although the number and intensities of signals were reduced (Fig. 2J-L and Fig. S1C). Very bright signals were also detected in oocytes hybridized with the antisense cyclin B1 RNA probe labeled with Fluorescein but not in oocytes hybridized with the sense cyclin B1 Fluorescein-labeled RNA probe (Fig. S1D and E), confirming that DIG- and Fluorescein-labeled RNA probes were similarly used in our method.

The differences in the signal intensities of *Pou5f1/Oct4*, *Emi2* and cyclin B1 mRNAs are consistent with the differences in the amounts of mRNAs, i.e., the amount of cyclin B1 mRNA in mouse oocytes is significantly larger than the amounts of *Emi2/Fbxo43* and *Pou5f1/Oct4* mRNAs [6, 37]. In addition, this method is highly reproducible because similar results were constantly obtained from five independent experiments (Fig. 2D, H, and L, see also Fig. S3). Taken together, we conclude that this method enables detection of the accumulation and distribution of mRNAs in mouse oocytes and embryos in a highly specific manner with almost no background. Furthermore, the procedure of this method is easy and requires small amounts of reagents, even compared with in situ hybridization using paraffin-embedded Sections [23], because all of the steps are performed using 24- and 96-well plates (Fig. 1B).

### Analysis of the Properties of *Pou5f1/Oct4* RNA Granules

**Fig. 3:**
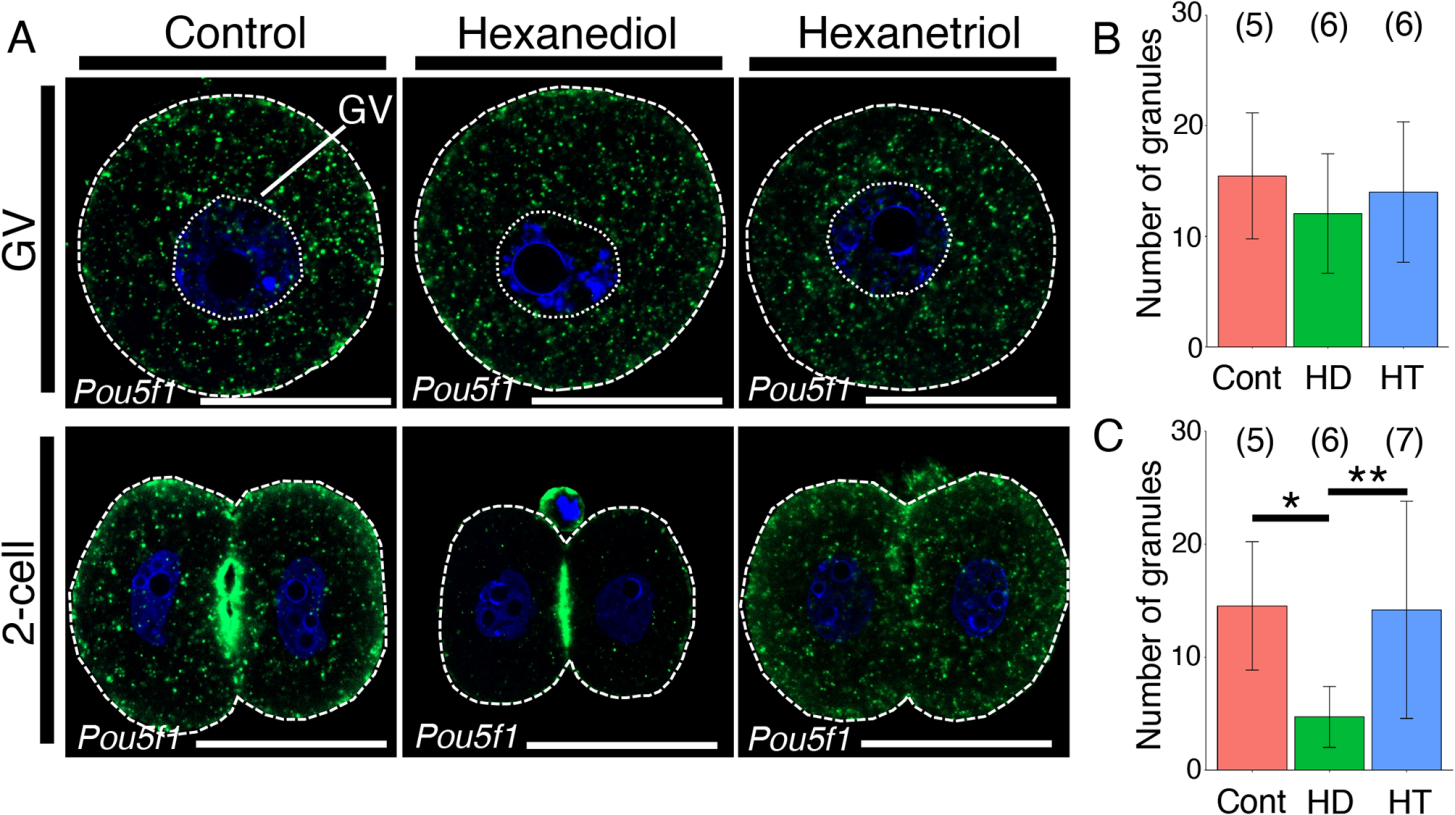
in situ hybridization of *Pou5f1/Oct4* mRNA in mouse oocytes and embryos treated with hexanediol or hexanetriol. **A** Detection of *Pou5f1/Oct4* mRNA (green) in immature oocytes (GV) and 2-cell stage embryos (2-cell) without (Control) and with hexanediol or hexanetriol. The oocytes and embryos are outlined by broken lines. DNA is shown in blue. Similar results were obtained from two independent experiments. GV, germinal vesicle. Bars, 50 μm. **B** The number of RNA granules per 100 µm^2^ in immature oocytes was counted (means ± standard deviations). **C** The number of RNA granules per 100 µm^2^ in 2-cell stage embryos was counted. The numbers in parentheses indicate the total numbers of oocytes and embryos analyzed. Statistical significance was analyzed by the Tukey-Kramer test. *p < 0.05, **p < 0.01. Cont, without treatment; HD, treated with hexanediol; HT, treated with hexanetriol

Our previous study demonstrated that translationally repressed *pou5f3* mRNA, which encodes Pou5f3, a homolog of Pou5f1/Oct4, was stored in zebrafish oocytes as RNA granules in a solid-like state [26]. These RNA granules changed into liquid-like droplets shortly after fertilization. We then analyzed the properties of *Pou5f1/Oct4* RNA granules by treating mouse oocytes and embryos with 50 mg/ml hexanediol, which dissolves assemblies in a liquid-like state but does not affect assemblies in a solid-like state [38], for 20 min at 37˚C. As a control, oocytes and embryos were treated with 50 mg/ml hexanetriol, a less effective chemical [38], for 20 min at 37˚C. Whole-mount in situ hybridization of oocytes showed that *Pou5f1/Oct4* RNA granules were not altered by treatment with hexanediol or hexanetriol (Fig. 3A and B). In contrast, *Pou5f1/Oct4* RNA granules were dissolved by treatment with hexanediol, but not by treatment with hexanetriol, in 2-cell stage embryos (Fig. 3A and C). The numbers of mitochondria and Golgi apparatus were not changed by treatment with hexanediol both in oocytes and 2-cell stage embryos (Fig. S4A-F). These results confirmed that hexanediol specifically dissolved assemblies in a liquid-like state and that the dissolution of *Pou5f1/Oct4* RNA granules was not indirectly caused by disassembly of these organelles. Similar results were obtained for all oocytes and embryos in two independent experiments. Taken together, the results suggest that *Pou5f1/Oct4* mRNA assembles into solid-like granules in oocytes and that the property is changed into a liquid-like state in embryos as in the case of zebrafish *pou5f3* mRNA.

### High-resolution analysis of cyclin B1 and *Emi2* mRNAs

**Fig. 4:**
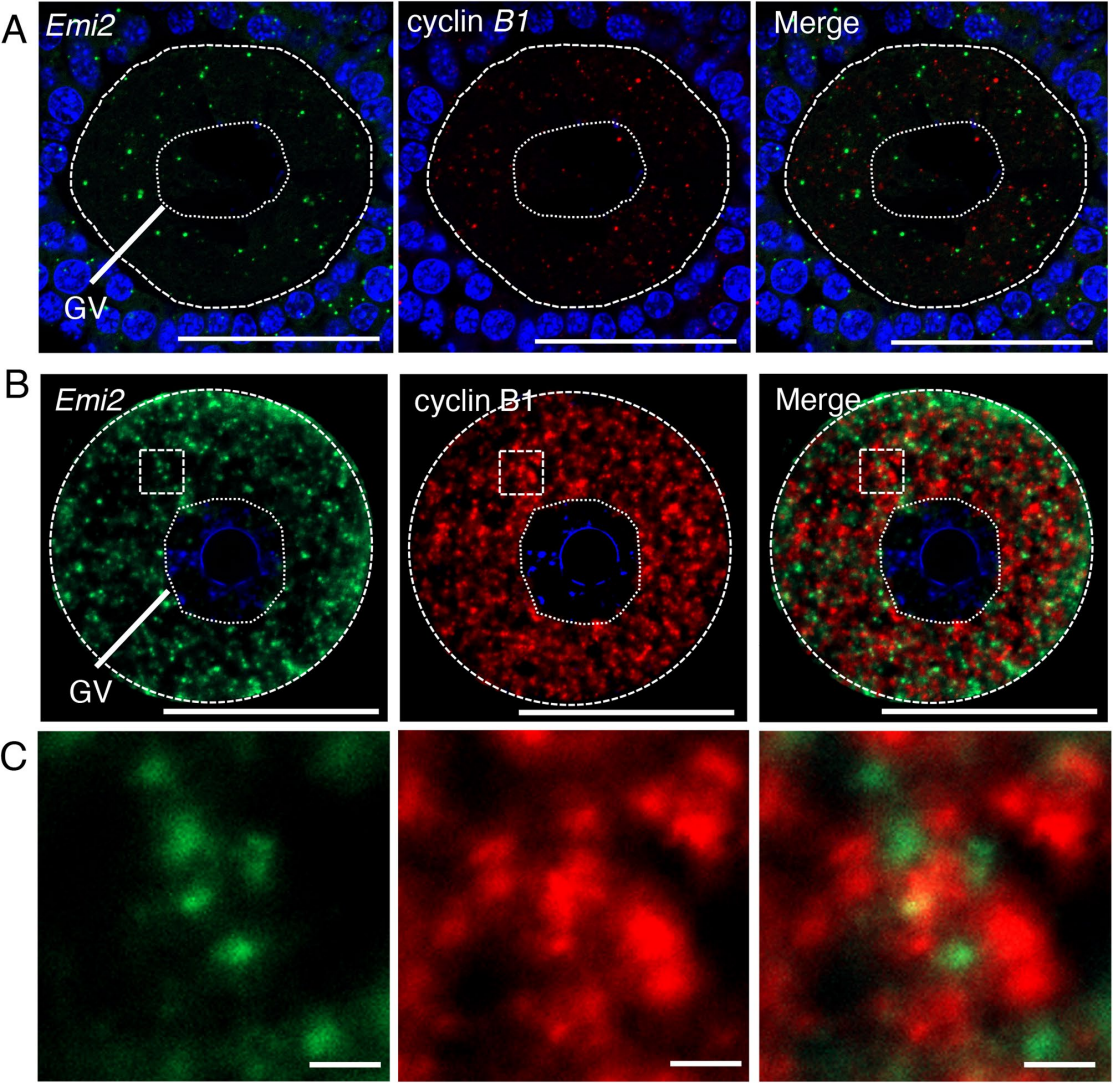
Double fluorescence in situ hybridization of *Emi2* (green) and cyclin B1 (red) mRNA in mouse oocytes. **A** Detection of *Emi2* (left) and cyclin B1 (middle) mRNAs by section in situ hybridization. A merged image is shown (Merge). DNA is shown in blue. Note that the signals in granulosa cells would be background of the TSA system in the section in situ hybridization method, which was caused by strong signal amplification, because similar signals were also observed in control sections hybridized with the *Emi2* sense RNA probe. **B** Detection of *Emi2* (left) and cyclin B1 (middle) mRNAs by whole-mount in situ hybridization. A merged image is shown (Merge). DNA is shown in blue. **C** Enlarged views of the boxed region in (B). Similar results were obtained from two independent experiments. GV, germinal vesicle. Bars, 50 μm in A and B, 1 μm in C

We then tested whether this whole-mount in situ hybridization method was applicable to the detection of different mRNAs simultaneously in the same sample. We first performed double in situ hybridization of cyclin B1 and *Emi2* mRNAs by hybridizing mouse oocytes with the Fluorescein-labeled cyclin B1 RNA probe and the DIG-labeled *Emi2* RNA probe. As observed in our previous study [13], section in situ hybridization showed that both mRNAs were distributed by forming different RNA granules (Fig. 4A). The whole-mount in situ hybridization method detected both mRNAs simultaneously in the same oocyte (Fig. 4B) and, remarkably, the intensities of the signals were significantly high compared with those in section in situ hybridization. The granular structures of cyclin B1 mRNA and *Emi2* mRNA were observed in enlarged views of images (Fig. 4C). We found that the sizes of cyclin B1 RNA granules were larger than those of *Emi2* RNA granules and that *Emi2* RNA granules were localized in the spaces between cyclin B1 RNA granules (Fig. 4C and Fig. S5A). Consistent with observations in our previous study [13], granules of *Emi2* mRNA and cyclin B1 mRNA were rarely co-localized in the cytoplasm of immature oocytes (Fig. 4B and C). Knockdown of *Emi2* mRNA by siRNA resulted in a significant reduction of *Emi2* signals without affecting cyclin B1 signals (Fig. S6A-C), confirming the specificity of signals and no cross-reaction of the probes. Similar results were obtained for all oocytes in two independent experiments (see also Fig. S5B). Taken together, the results indicate that the whole-mount in situ hybridization method can simultaneously detect different mRNAs with high sensitivity and high resolution.

### Super-resolution analysis of *Pou5f1/Oct4* and Cyclin B1 mRNAs

**Fig. 5:**
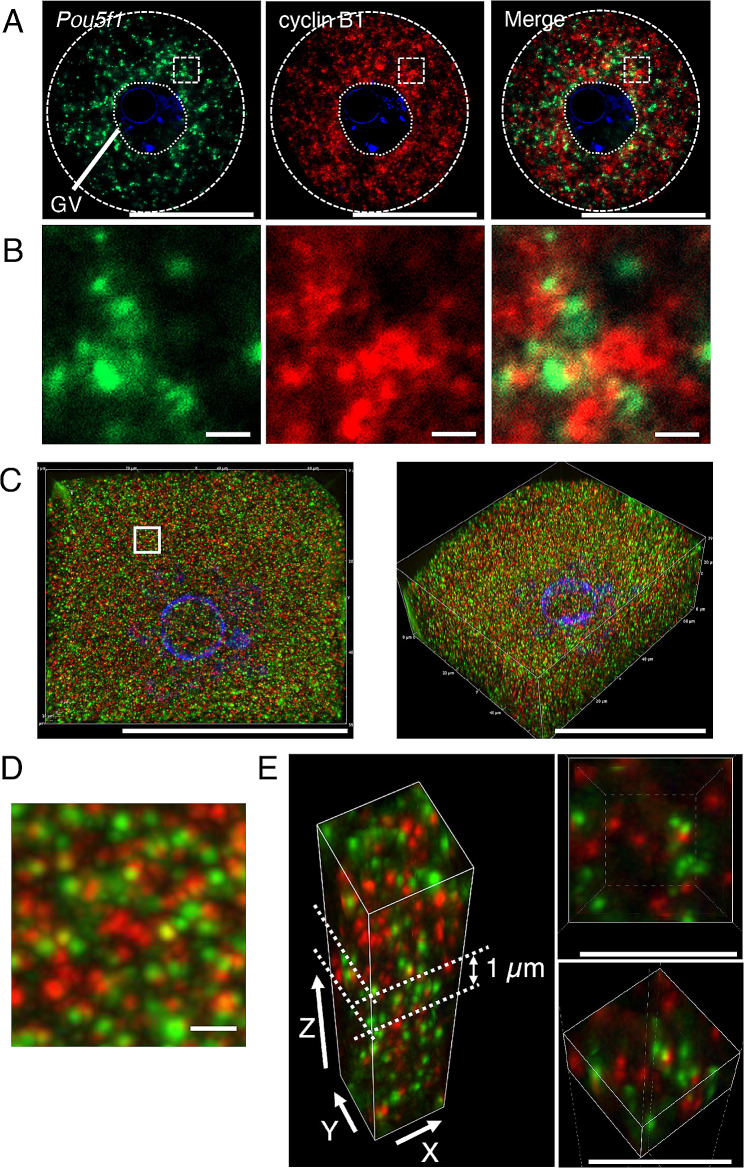
Double fluorescence in situ hybridization of *Pou5f1/Oct4* (green) and cyclin B1 (red) mRNAs in mouse oocytes. **A** Detection of *Pou5f1/Oct4* (left) and cyclin B1 (middle) mRNAs in an immature oocyte by whole-mount in situ hybridization. A merged image is shown (Merge). DNA is shown in blue. **B** Enlarged views of the boxed regions in (A). **C** Views of *Pou5f1/Oct4* and cyclin B1 mRNAs in an immature oocyte by super-resolution microscopy. A representative top view (X-Y view) is shown on the left. A 3D view (X-Y-Z view) is shown on the right. **D** An enlarged view of the 25 µm^2^ square region in (C). **E** Views of 3D reconstruction of the 25 µm^2^ square region in (C). Insets are a top view (upper) and a 3D view (lower) of the x-y axis of the 1-µm-thick z-stacks indicated. Similar results were obtained from two independent experiments. Bars, 50 μm in A and C, 1 μm in B, D and E

To investigate the localization of *Pou5f1/Oct4* and cyclin B1 mRNAs in mouse oocytes, we then performed double in situ hybridization of *Pou5f1/Oct4* and cyclin B1 mRNAs in immature oocytes. Consistent with the observations in single in situ hybridization (Fig. 2B and Fig. S1A), *Pou5f1/Oct4* RNA granules were detected in the oocyte cytoplasm and were mainly localized in the area around the GV (Fig. 5A). cyclin B1 RNA granules were distributed throughout the oocyte cytoplasm (Fig. 5A). Enlarged views showed that *Pou5f1/Oct4* and cyclin B1 RNA granules were distributed as different granules and that their sizes seemed to be varied (Fig. 5B). As in the case of cyclin B1 and *Emi2* mRNAs, distinct RNA granules were rarely co-localized. Knockdown of *Pou5f1/Oct4* mRNA by siRNA resulted in a reduction of *Pou5f1/Oct4* signals without affecting cyclin B1 signals (Fig. S6D-F), confirming the specificity of signals and no cross-reaction of the probes. Similar results were obtained for all oocytes in two independent experiments (see also Fig. S5C).

**Fig. 6:**
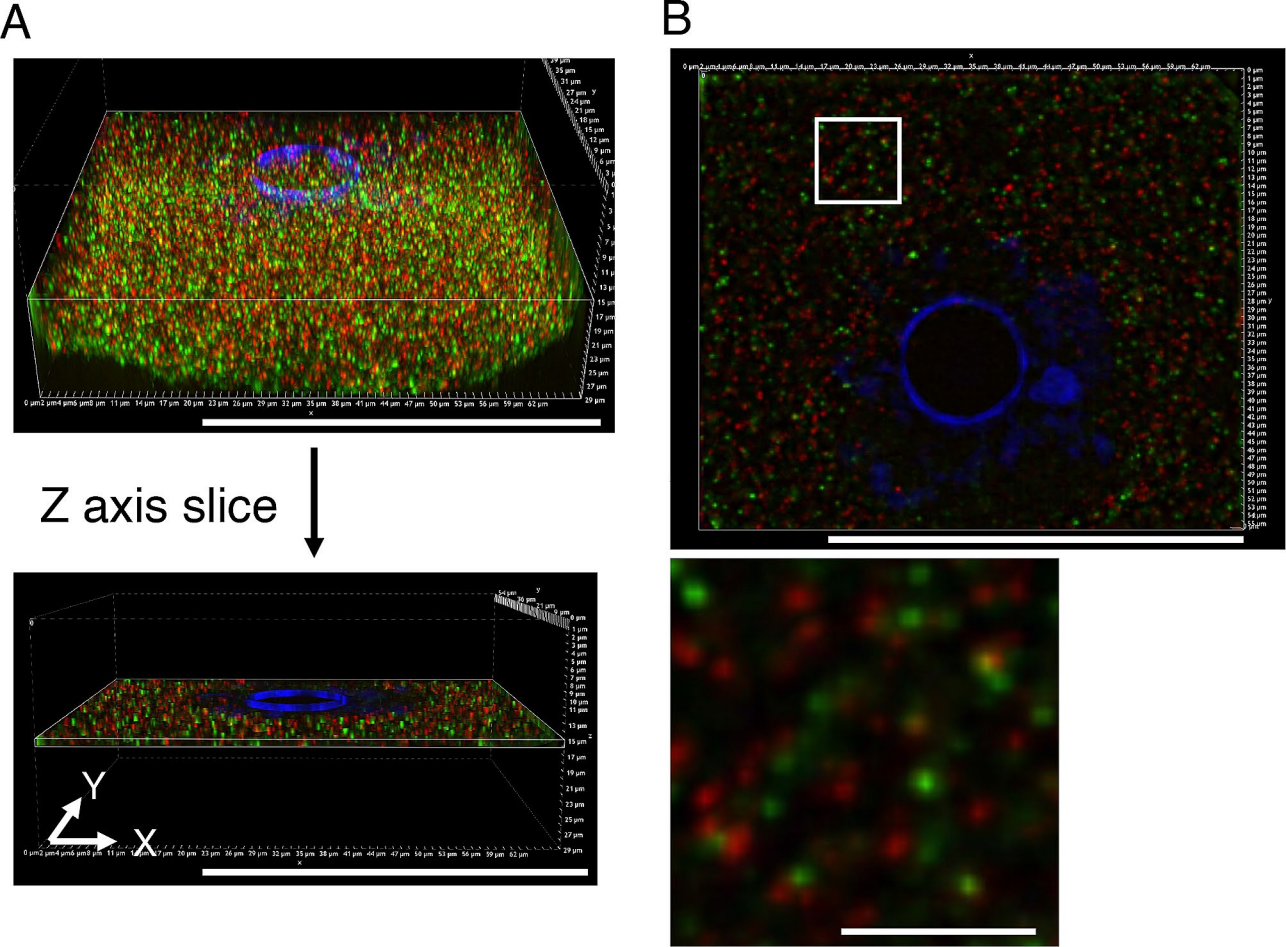
Reconstruction views of the x-y axis of 1-µm-thick z-stacks of a super-resolution microscope image. **A** Acquiring a reconstruction image of the x-y axis in a whole oocyte. One µm of z-stacks was trimmed from the whole oocyte image and analyzed. **B** Top view of the reconstructed image in (A) (upper) and enlarged view of the 100 µm^2^ square region in the upper image (lower). Bars, 50 μm in A and B (upper), 5 μm in B (lower)

**Fig. 7:**
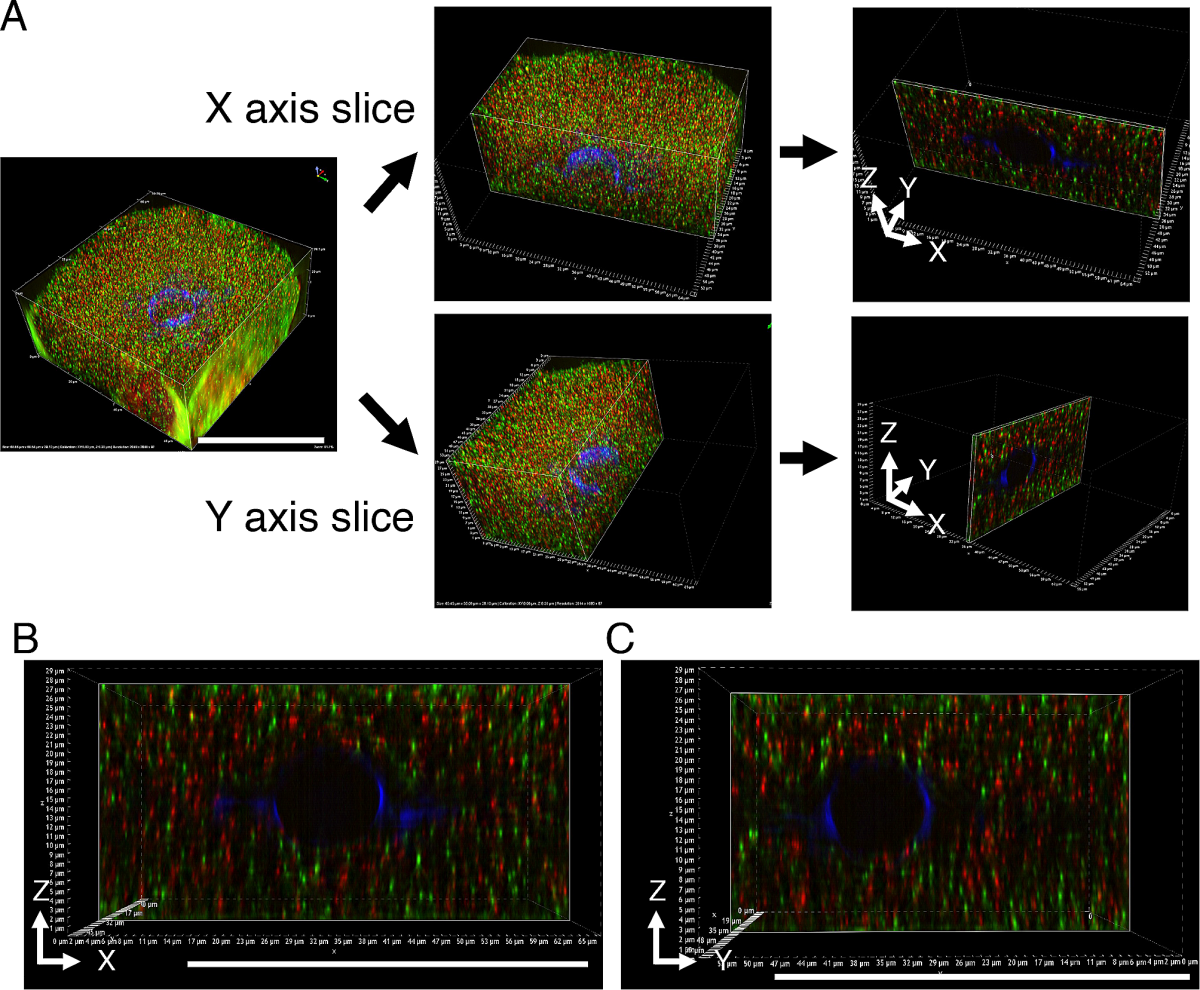
Reconstruction views of x-z and y-z axes of super-resolution microscope images. **A** Acquiring a reconstruction image of x-z and y-z axes in the whole oocyte. One µm y- or x-stacks was trimmed from the whole oocyte images. **B** X-Z view of the reconstructed image in (A, upper). **C** Y-Z view of the reconstructed image in (A, lower). Bars, 50 μm

To examine the relationship between *Pou5f1/Oct4* and cyclin B1 mRNAs in more detail, mouse oocytes were observed using structured illumination microscopy (SIM), which allows a two-fold higher resolution than that of conventional confocal microscopy. SIM enables observation of signals with multiple fluorophores used in conventional confocal microscopy without labeling with special fluorophores, although the resolution is limited to ~ 120 nm. We constructed 3-dimensional (3D) distributions of the mRNAs in almost whole oocytes by taking 30-µm-thick z-stacks at 0.3-µm intervals (Fig. 5C). We first analyzed a 25 µm^2^ square region of z-stacks (boxed region in Fig. 5C). Although a single projection image of this region showed that some *Pou5f1/Oct4* and cyclin B1 RNA granules were overlapped (Fig. 5D), analysis of the 3D reconstruction of the same image indicated that these RNA granules were not co-localized (Fig. 5E). We expanded this analysis to the whole oocyte. In analysis of the x-y axis of 1-µm-thick z-stacks, co-localization of *Pou5f1/Oct4* and cyclin B1 RNA granules was not observed (Fig. 6). Similar results were obtained in the analysis of reconstruction in x-z and y-z axes (Fig. 7). Moreover, this super-resolution analysis demonstrated that *Pou5f1/Oct4* and cyclin B1 mRNAs were distributed as granules of similar sizes (Figs. 5C-E, 6 and 7). RNA granules consisting of *Pou5f1/Oct4* mRNA were frequently localized close to each other. A similar localization pattern was observed in RNA granules consisting of cyclin B1 mRNA. This analysis is highly reproducible because similar results were obtained in four oocytes of two independent experiments. In addition, our in situ hybridization method allows detection of whole signals in thick samples (30 μm in depth) without photobleaching possibly due to high levels of signal intensities.

## Discussion

Oocytes deposit almost all molecules required for their maturation and the progression of early developmental processes. Because transcription is quiescent after initiation of oocyte maturation until mitotic cleavage stages, the proper accumulation of thousands of mRNAs is fundamental for oocytes to obtain competence to promote maturation and early development after fertilization. However, detailed analysis of the accumulation of individual mRNAs in mammalian oocytes has been limited due to their small sizes and instability in handling them. We developed a whole-mount in situ hybridization method with high sensitivity and super-resolution. This method enables analysis of the mRNA accumulation in a simple, convenient and cost-effective manner compared with section in situ hybridization combined with the TSA system [23] and whole-mount smRNA-FISH with the RNA-scope technique [27–29]. For example, the amounts of reagents required for this method are approximately 1,000-times smaller than the amounts of reagents required for section in situ hybridization, in which 100–200 ml of reagents are required for each step, and this method is possibly several times less costly than smRNA-FISH with the RNA-scope, which requires Z probes, three round hybridization steps, and a probe-labeling step with commercially available kits. The procedure can be completed within 3 days, which is 2–3 days less than section in situ hybridization because of omission of the paraffin-embedding and section-making steps.

Using this method, we could clearly detect the distribution patterns of *Pou5f1/Oct4*, *Emi2* and cyclin B1 mRNA molecules in mouse oocytes and 2-cell stage embryos (Fig. 2). Although we previously found that these mRNAs form granular structures in mouse oocytes ([13, 21, 23–25], see also Fig. 4A), the newly established method for whole-mount in situ hybridization showed more accurate mRNA distributions due to its higher sensitivity than that of section in situ hybridization. In addition, we could detect *Pou5f1/Oct4* RNA granules in 2-cell stage embryos (Figs. 2C and 3A), which were not detected in section in situ hybridization. Moreover, this method enabled easy examination of distribution patterns of mRNAs in isolated oocytes and embryos after treatment with any chemicals or injection of any materials. Indeed, we found that *Pou5f1/Oct4* mRNA was assembled as solid-like granules in oocytes, whereas the property was changed into liquid-like droplets in 2-cell stage embryos by treating oocytes and embryos with hexanediol (Fig. 3). Our previous studies demonstrated translational activation of this mRNA in early 2-cell stage mouse embryos [25, 39]. Thereby, it is likely that the translation of *Pou5f1/Oct4* mRNA is activated in granules in 2-cell stage embryos as in the case of embryonic RNA granules found in zebrafish [26], although the translational state of *Pou5f1/Oct4* mRNA in the liquid-like droplets should be examined. In this regard, it is of interest that the properties of cyclin B1 RNA granules are also different in oocytes and embryos and that they act as translational sites in embryos.

The distribution patterns of *Pou5f1/Oct4* and cyclin B1 mRNAs were further analyzed by using the SIM super-resolution microscope. An intriguing finding in this analysis is that almost all of the RNA granules are distributed in similar sizes (Figs. 5C-E, 6 and 7). This is different from the observations using a confocal microscope, in which cyclin B1 and *Pou5f1/Oct4* mRNAs seemed to be assembled into granules of different sizes (Fig. 5A and B). A possible explanation for this difference is that the super-resolution analysis (~ 120 μm) revealed the existence of basic-size units of RNA granules in oocytes. Assembly of these basic-size units close to each other would be observed as large-sized granules in confocal microscopy. Another intriguing finding in the super-resolution analysis is that *Pou5f1/Oct4* RNA granules are not co-localized with cyclin B1 RNA granules, suggesting that distinct mRNAs are assembled into homotypic granules and that granules consisting of the same mRNA tend to be assembled close to each other. Further, the results suggest the mechanisms by which distinct mRNAs are spatially regulated in the oocyte cytoplasm after transcription in the nucleus. Although it had been thought that mRNA molecules are evenly distributed throughout the cell cytoplasm, technical advances in the detection of RNAs have revealed spatial control of mRNAs by localizing and/or forming granular structures (see reviews [40–42]). The results of this study provide more insights into the molecular nature of mRNA regulation in oocytes: basic-sized homotypic RNA granules are assembled into clusters, possibly resulting in compartmentalization of the oocyte cytoplasm. This spatial control of mRNAs should be linked to the temporal control of mRNA translation.

Then, how are these distribution patterns of distinct mRNAs regulated? Extensive studies have demonstrated that *cis*-acting elements in mRNAs are recognized by *trans*-acting factors such as RNA-binding proteins, leading to the spatial and temporal control of mRNA localization and translation [40–42]. To date, some RNA-binding proteins have been identified as *trans*-acting factors of *Pou5f1/Oct4*, *Emi2* and cyclin B1 mRNAs [13, 24, 39, 43, 44]. We previously demonstrated that pumilio1 protein formed assemblies and co-localized with cyclin B1 RNA granules in mouse oocytes by section in situ hybridization combined with immunofluorescence [24]. In contrast, Tdrd3 formed assemblies and co-localized with *Emi2* RNA granules [13]. The method for whole-mount in situ hybridization established in this study will facilitate studies on the regulation of mRNAs by *trans*-acting factors because this method can detect accurate distribution patterns of mRNAs due to its high sensitivity and proteins can be easily detected due to the omission of proteinase treatment and paraffin embedding from the procedure. Our preliminary data demonstrated the co-localization of *Pou5f1/Oct4* RNA granules with hnRNP D protein, which was identified as a protein binding to *Pou5f1/Oct4* mRNA [39], in oocytes, suggesting that mRNAs and proteins are indeed simultaneously detected in this method. In addition, RNA probes for many mRNAs can be easily prepared using plasmid vectors containing genes of interest in a cost-effective manner, and three or four different mRNAs can be detected in single oocytes and embryos by labeling RNA probes with DIG, Fluorescein, dinitrophenyl (DNP), and biotin. It will be of interest to determine how large complexes of mRNAs-proteins are involved in translational control and how distinct mRNAs are translated at the appropriate times and places.

Our in situ hybridization method would be applicable to preimplantation mammalian embryos including those in mice and humans, which will facilitate studies on early development of mammals. However, this method is not applicable to later stage embryos and adult organisms, which require histological sections. In addition, although this method enabled the detection of some mRNAs in mouse oocytes and early-stage embryos, it remains unclear whether all of the mRNAs that have accumulated in oocytes and embryos can be detected. Recent technical advances have enabled the detection of translation sites of particular mRNAs in oocytes and embryos based on the proximity-ligation assay using two antibodies derived from different species [26, 28, 45]. It is technically possible but still challenging that detection of direct mRNA-protein binding using the proximity-ligation assay. The newly established whole-mount in situ hybridization method can be combined with these assays. Taken together, the results indicate that this method is useful for studying the spatial and temporal regulation of mRNAs, which is important for oocytes to drive maturation and embryonic development.

### Electronic Supplementary Material

Below is the link to the electronic supplementary material.

Supplementary Material 1

## Data Availability

No datasets were generated or analysed during the current study.
