# Supplementary material for: High-sensitivity whole-mount in situ Hybridization of Mouse Oocytes and Embryos Visualizes the Super-resolution Structures and Distributions of mRNA Molecules
Source: Biol Proced Online. 2024 Jul 10;26:23. doi: 10.1186/s12575-024-00250-5 (PMC11234658; doi:10.1186/s12575-024-00250-5)
Supplement: Supplementary file 1 — Supplementary Material 1 [file 12575_2024_250_MOESM1_ESM.pdf]

Supplemental materials and methods

Whole-mount immunofluorescence

To detect mitochondria and the Golgi apparatus, mouse oocytes and embryos were immunostained with anti-mtTFA and anti-GM130 antibodies, respectively. Briefly, mouse oocytes and embryos were treated with 1,6-hexanediol or 1,2,6-hexanetriol for 20 min at 37˚C and were fixed with 4% PFA for 30 min at room temperature. After permeabilization with 0.1% Triton-X100 in PBS for 20 min, the samples were incubated with blocking solution (0.3% BSA, 0.01% Tween-20 in PBS) for 1 h at room temperature and then incubated with anti-mtTFA antibody (1:200; Abcam; ab47517) or anti-GM130 antibody (1:200; BD Transduction Laboratories; 610822) overnight at 4°C. The samples were washed with blocking solution three times and then incubated with Alexa Flour 488-conjugated anti-rabbit or mouse IgG secondary antibody (Molecular Probes). The samples were mounted with VECTASHIELD Mounting Medium with DAPI and observed under the LSM 980 confocal microscope.

Supplemental figure legends

Fig. S1 Quantitative analysis of *Pou5f1/Oct4*, *Emi*2 and cyclin B1 RNA granules in GV-stage oocytes and 2-cell stage embryos. **A** Distributions of *Pou5f1/Oct4*. *Emi2* and cyclin B1 RNA granules in inner (I) and outer (O) regions were analyzed (means ± standard deviations). The image on the left is a representative image with depiction of quantified areas. Statistical significance was analyzed by Student's *t*-test. *p<0.05. **B** The numbers (left) and intensities (right) of *Pou5f1/Oct4*, *Emi2* and cyclin B1 RNA granules in GV-stage oocytes were analyzed. Results from three independent experiments were summarized. Statistical significance was analyzed by the Tukey-Kramer test. ***p<0.001. **C** The intensities of cyclin B1 RNA granules in GV-stage oocytes (GV) and 2-cell stage embryos (2-cell) were analyzed. Results from three independent experiments were summarized. Statistical significance was analyzed by Student's *t*-test. ***p<0.001. **D-E** Single *in situ* hybridization of cyclin B1 mRNA in immature oocytes hybridized with the fluorescein (FITC)-labeled probe. Detection of cyclin B1 mRNA hybridized with the sense (A) or antisense (B) RNA probe. DNA is shown in blue. GV, germinal vesicle. Bars, 50 µm.

Fig. S2 Knockdown of *Pou5f1/Oct4*, *Emi2* or cyclin B1 mRNA by siRNA. **A** Sequences

of *Pou5f1/Oct4*, *Emi2* and cyclin B1 mRNAs that are targeted by *Pou5f1/Oct4*, *Emi2* and cyclin B1 siRNAs. **B-D** Detection of *Pou5f1/Oct4* mRNA in control non-injected oocytes (B-C) and oocytes injected with *Pou5f1/Oct4* siRNA (D) hybridized with the sense (B) or antisense (C-D) RNA probe. DNA is shown in blue. **E** The numbers of *Pou5f1/Oct4* RNA granules per 100 μm^2 in individual oocytes were counted (means ± standard deviations). Results from two independent experiments were summarized. **F-H** Detection of *Emi2* mRNA in control non-injected oocytes (F-G) and oocytes injected with *Emi2* siRNA (H) hybridized with the sense (F) or antisense (G-H) RNA probe. DNA is shown in blue. **I** The numbers of *Emi2* RNA granules per 100 μm^2 in individual oocytes were counted. Results from two independent experiments were summarized. **J-L** Detection of cyclin B1 mRNA in control non-injected oocytes (J-K) and oocytes injected with cyclin B1 siRNA (L) hybridized with the sense (J) or antisense (K-L) RNA probe. DNA is shown in blue. **M** The numbers of cyclin B1 RNA granules per 100 μm^2 in individual oocytes were counted. Results from two independent experiments were summarized. The numbers in parentheses indicate the total numbers of oocytes and embryos analyzed. Statistical significance was analyzed by Student's *t*-test. ***$p<0.001$. Bars, 50 μm.

Fig. S3 *In situ* hybridization of *Pou5f1/Oct4*, *Emi2* and cyclin B1 mRNAs in mouse oocytes and embryos. **A-B** Results of two experiments independent from those for which results are shown in Fig. 2. Immature oocytes and 2-cell stage embryos were hybridized with the sense (S) or antisense (AS) RNA probes. DNA is shown in blue. Bars, 50 μm.

Fig. S4 Immunofluorescence of mitochondria and the Golgi apparatus in oocytes and embryos treated with hexanediol or hexanetriol. **A** Detection of mitochondria with anti-mtTFA antibody (green) in immature oocytes (GV) and 2-cell stage embryos (2-cell) without (Control) and with hexanediol or hexanetriol. DNA is shown in blue. **B** The number of mitochondria per 100 μm^2 in immature oocytes was counted (means ± standard deviations). **C** The number of RNA granules per 100 μm^2 in 2-cell stage embryos was counted. **D** Detection of the Golgi apparatus with anti-GM130 antibody (green) in immature oocytes and 2-cell stage embryos without and with hexanediol or hexanetriol. DNA is shown in blue. **E** The number of Golgi apparatus per 100 μm^2 in

immature oocytes was counted. **F** The number of Golgi apparatus per 100 μm^2 in 2-cell stage embryos was counted. The numbers in parentheses indicate the total numbers of oocytes and embryos analyzed. Statistical significance was analyzed by the Tukey-Kramer test. Cont, without treatment; HD, treated with hexanediol; HT, treated with hexanetriol. Similar results were obtained from two independent experiments. Bars, 50 μm.

Fig. S5 Double fluorescence *in situ* hybridization of cyclin B1 mRNA and *Emi2* or *Pou5f1/Oct4* mRNA in mouse oocytes. **A** Quantitative analysis of *Emi2* and cyclin B1 RNA granules. The sizes of *Emi2* and cyclin B1 RNA granules in GV-stage oocytes were analyzed (means ± standard deviations). Results from three independent experiments were summarized. Statistical significance was analyzed by Student's *t*-test. **p<0.01. **B** Double fluorescence *in situ* hybridization of *Emi2* (green) and cyclin B1 (red) mRNA in mouse oocytes. A merged image is shown (Merge). Results of one experiment independent from the experiment for which results are shown in Fig. 4. **C** Double fluorescence *in situ* hybridization of *Pou5f1/Oct4* (green) and cyclin B1 (red) mRNA in mouse oocytes. A merged image is shown (Merge). Results of one experiment independent from the experiment for which results are shown in Fig. 5. DNA is shown in blue. Bars, 50 μm.

Fig. S6 Double fluorescence *in situ* hybridization of cyclin B1 mRNA and *Emi2* or *Pou5f1/Oct4* mRNA in mouse oocytes injected with *Pou5f1/Oct4* or *Emi2* siRNA. **A-B** Detection of *Emi2* (left) and cyclin B1 (middle) mRNAs by whole-mount *in situ* hybridization in oocytes not injected (A) or injected with *Emi2* siRNA (B). A merged image is shown (Merge). DNA is shown in blue. **C** The numbers of *Emi2* (upper) and cyclin B1 (lower) RNA granules per 100 μm^2 in individual oocytes were counted (means ± standard deviations). Results from two independent experiments were summarized. **D-F** Detection of *Pou5f1/Oct4* (left) and cyclin B1 (middle) mRNAs by whole-mount *in situ* hybridization in oocytes not injected (D) or injected with *Pou5f1/Oct4* siRNA (E). A merged image is shown (Merge). DNA is shown in blue. **F** The numbers of *Pou5f1/Oct4* (upper) and cyclin B1 (lower) RNA granules per 100 μm^2 in individual oocytes were counted. Results from two independent experiments were summarized. The numbers in parentheses indicate the total numbers of oocytes and

embryos analyzed. Statistical significance was analyzed by Student's *t*-test. ***$p<0.001$. Similar results were obtained from two independent experiments. Bars, 50 μm.

Fig. S1

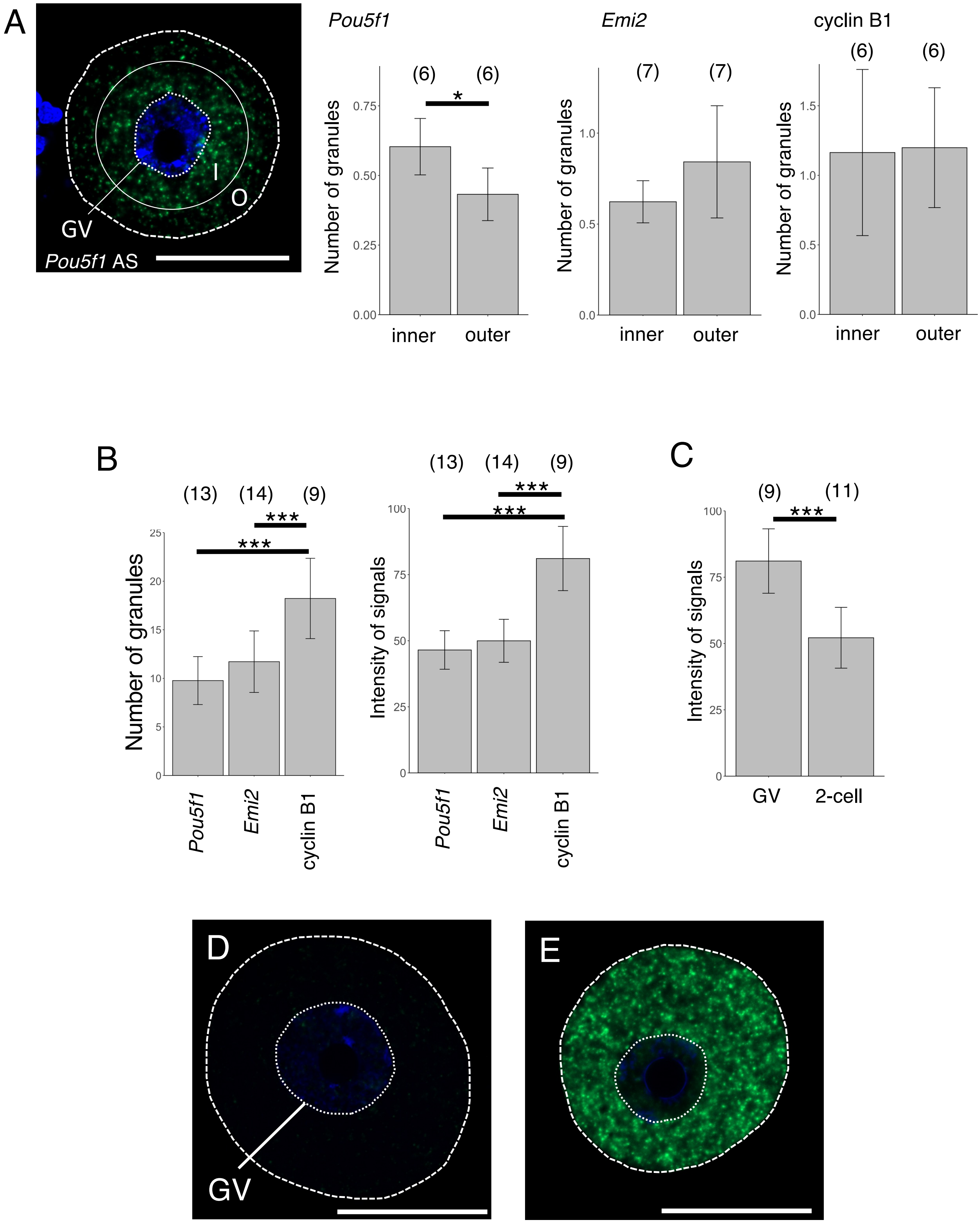
A
GV
I
O
Pou5f1 AS
Pou5f1
Emi2
cyclin B1
(6)
(7)
Number of granules
inner
outer
B
(13)
(14)
(9)
Intensity of signals
C
(11)
GV
2-cell
D
E

Fig. S2

A		
	Pou5f1	CAGCCUUAAGAACAUGUGUAAGC
	Emi2	CUGUCAGAAUCUUGCAGCCUUAAUA
	**cyclin B1**	UCGAAUUUAGGAGUAUAUUAAAA

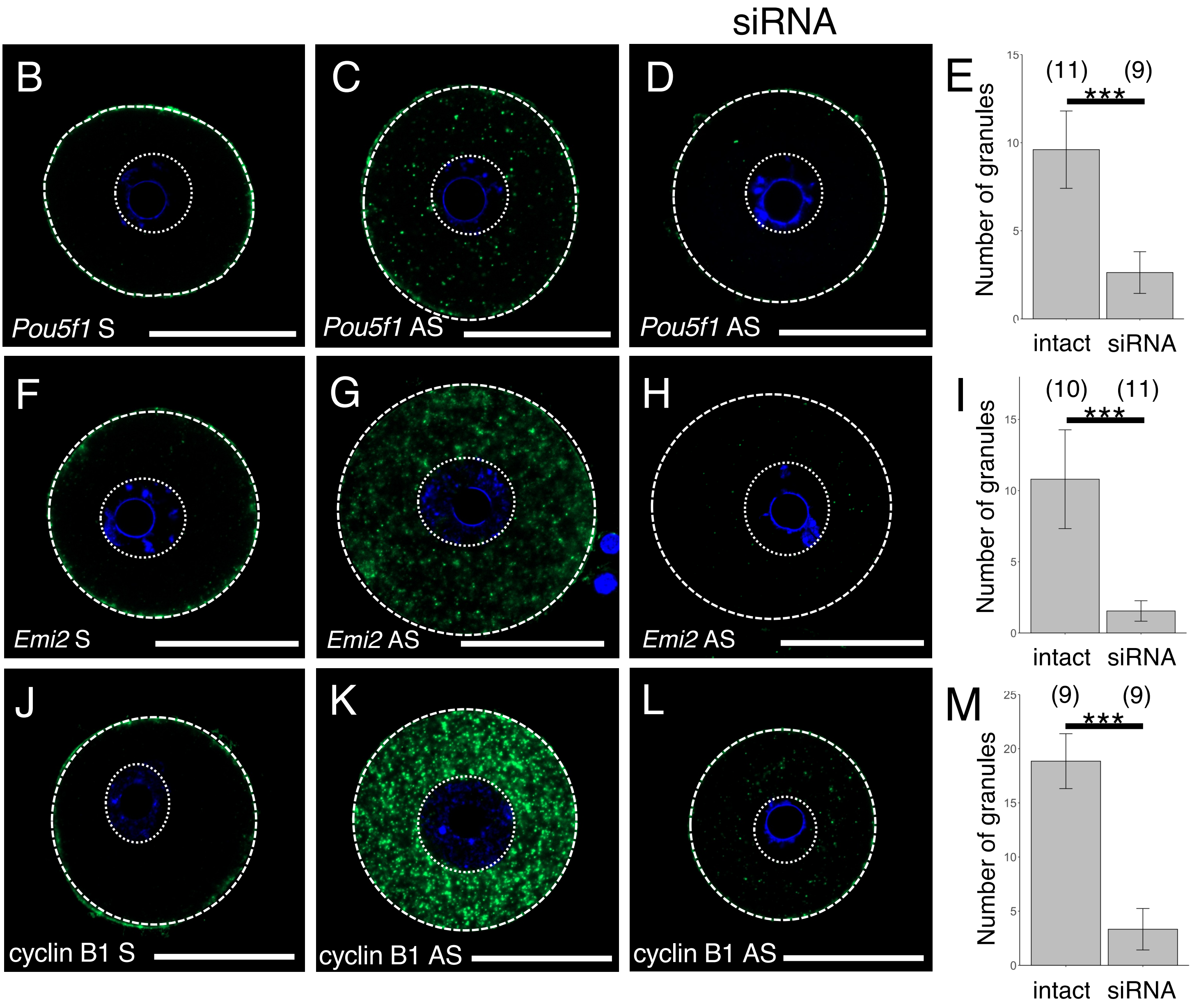

Fig. S3

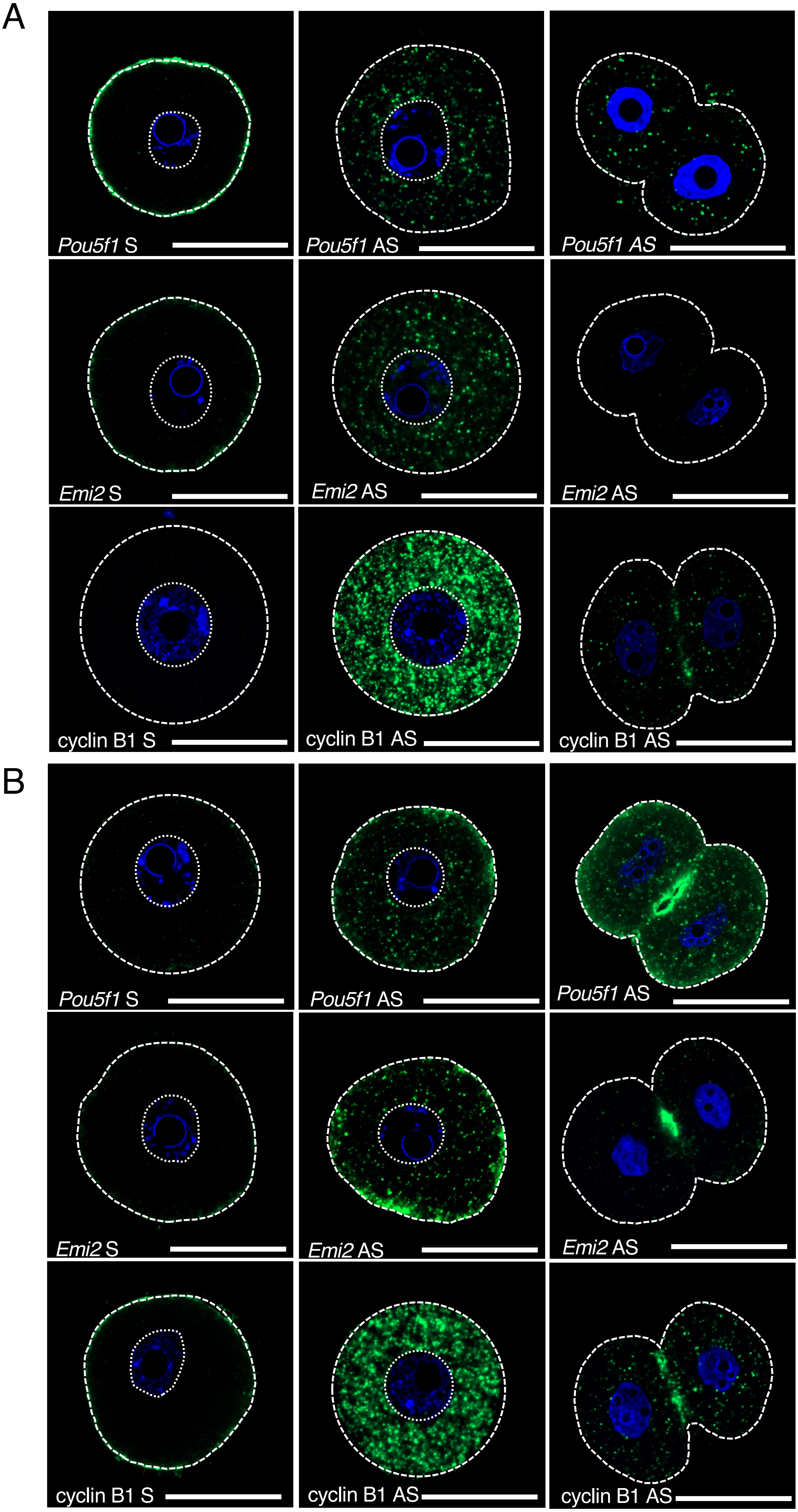
A
Pou5f1 S
Pou5f1 AS
Pou5f1 AS
Emi2 S
Emi2 AS
Emi2 AS
cyclin B1 S
cyclin B1 AS
cyclin B1 AS
B
Pou5f1 S
Pou5f1 AS
Pou5f1 AS
Emi2 S
Emi2 AS
Emi2 AS
cyclin B1 S
cyclin B1 AS
cyclin B1 AS

Fig. S4

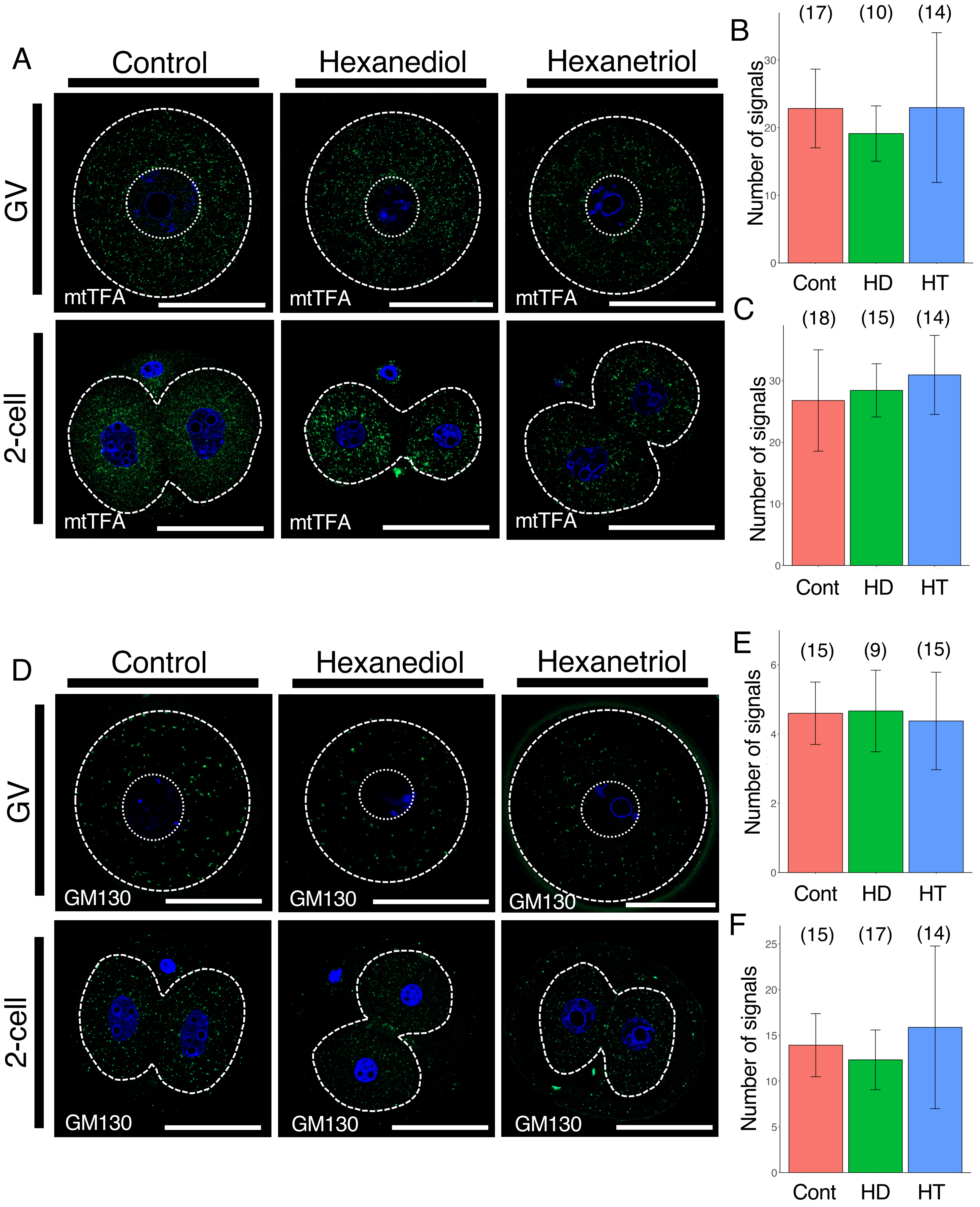
A
Control
Hexanediol
Hexanetriol
GV
2-cell
mtTFA
B
(17)
(10)
(14)
Number of signals
Cont
HD
HT
C
(18)
(15)
(14)
D
GM130
E
(15)
(9)
(15)
F
(15)
(17)
(14)

Fig. S5

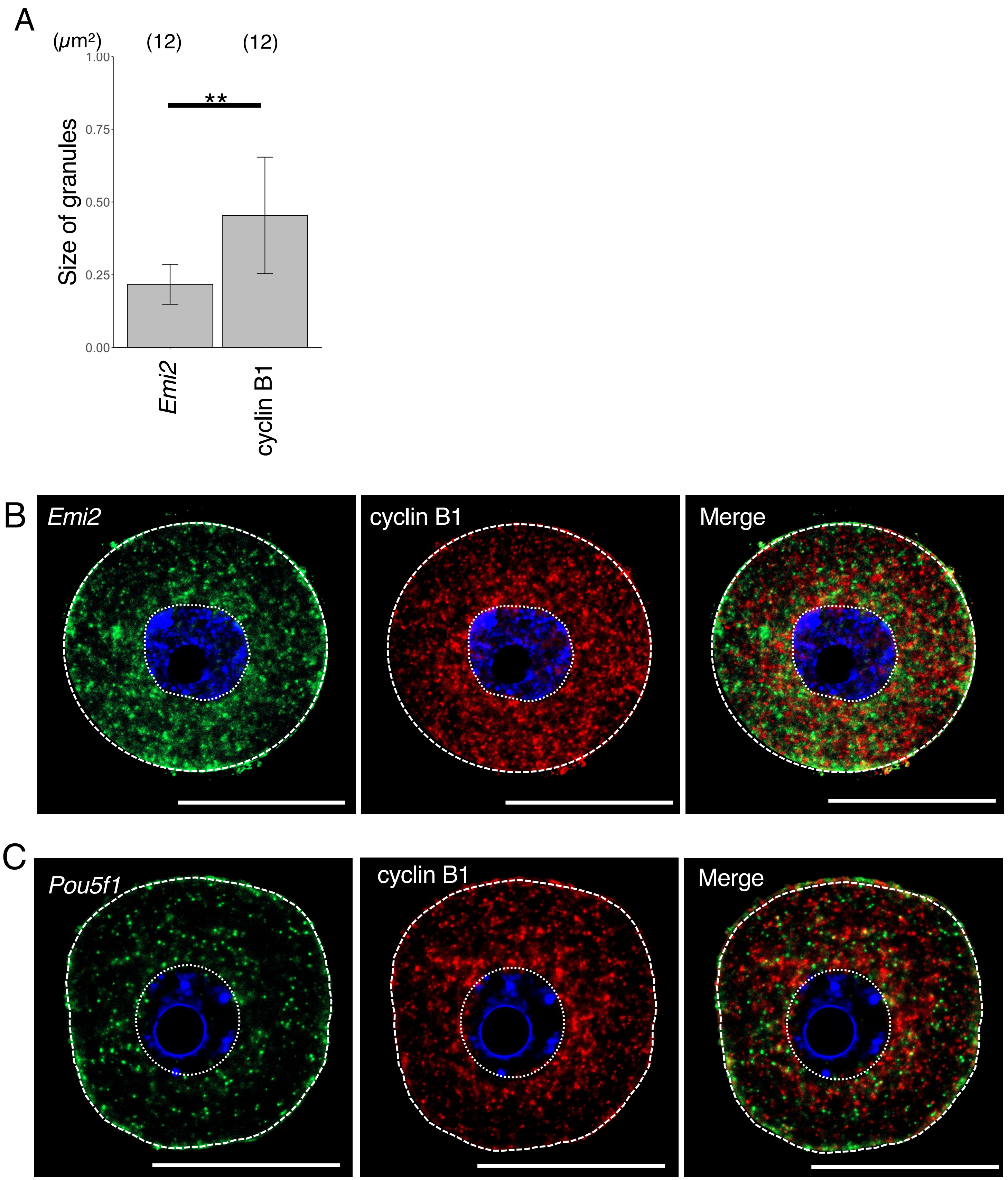
A
(μm2)
(12)
(12)
1.00
0.75
0.50
0.25
0.00
**
Size of granules
Emi2
cyclin B1
B
Emi2
cyclin B1
Merge
C
Pou5f1
cyclin B1
Merge

Fig. S6

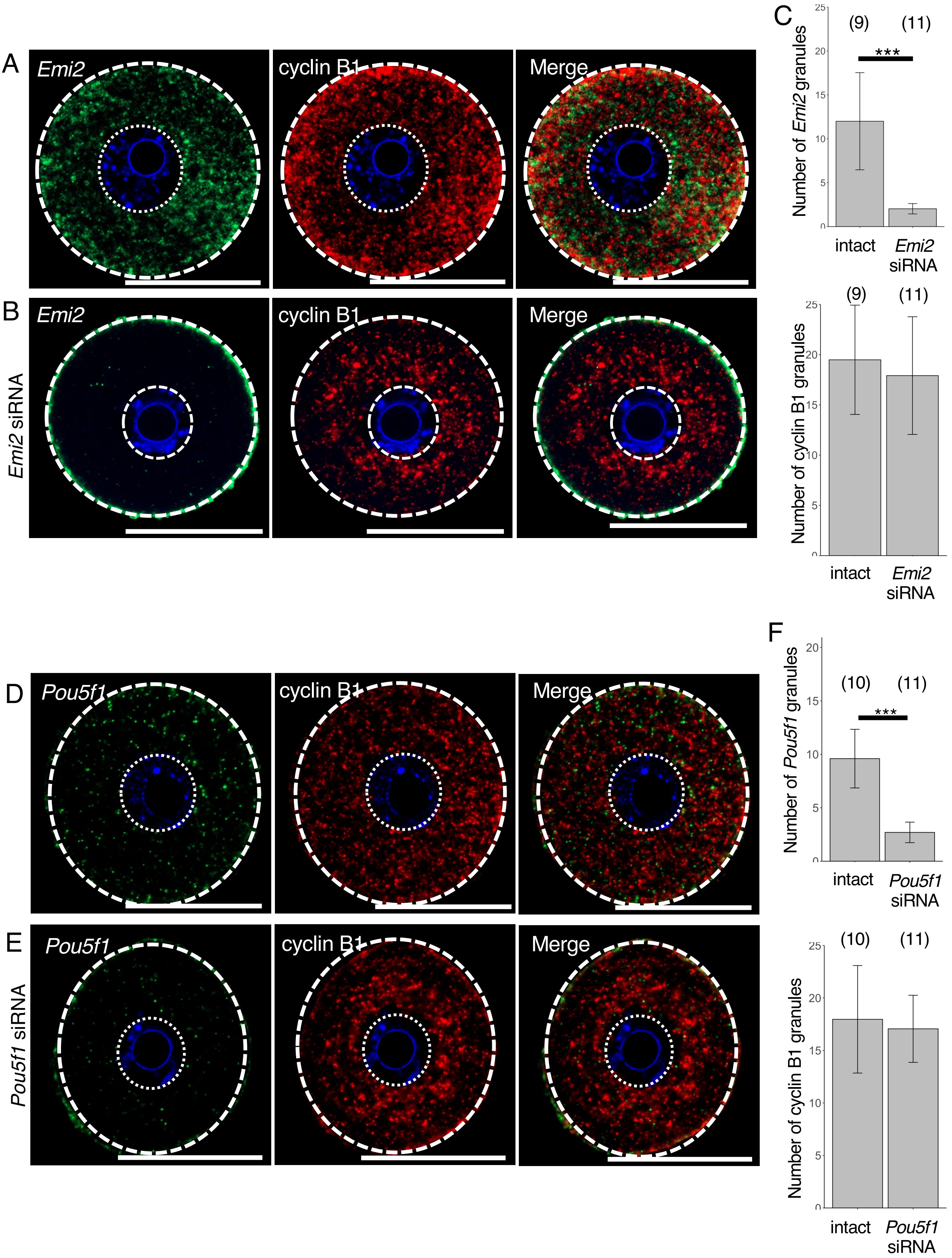

A
Emi2
cyclin B1
Merge
B
Emi2 siRNA
C
(9)
(11)
***
Number of Emi2 granules
intact
Emi2 siRNA
Number of cyclin B1 granules
D
Pou5f1
E
Pou5f1 siRNA
F
(10)
Number of Pou5f1 granules
Pou5f1 siRNA
